# Predicting the Mortality and Readmission of In-Hospital Cardiac Arrest Patients With Electronic Health Records: A Machine Learning Approach

**DOI:** 10.2196/27798

**Published:** 2021-09-13

**Authors:** Chien-Yu Chi, Shuang Ao, Adrian Winkler, Kuan-Chun Fu, Jie Xu, Yi-Lwun Ho, Chien-Hua Huang, Rohollah Soltani

**Affiliations:** 1 Department of Emergency Medicine Yunlin Branch National Taiwan University Hospital Yunlin Taiwan; 2 Knowtions Research Toronto, ON Canada; 3 Department of Internal Medicine National Taiwan University Hospital Taipei Taiwan; 4 Department of Emergency Medicine National Taiwan University Hospital Taipei Taiwan

**Keywords:** in-hospital cardiac arrest, 30-day mortality, 30-day readmission, machine learning, imbalanced dataset

## Abstract

**Background:**

In-hospital cardiac arrest (IHCA) is associated with high mortality and health care costs in the recovery phase. Predicting adverse outcome events, including readmission, improves the chance for appropriate interventions and reduces health care costs. However, studies related to the early prediction of adverse events of IHCA survivors are rare. Therefore, we used a deep learning model for prediction in this study.

**Objective:**

This study aimed to demonstrate that with the proper data set and learning strategies, we can predict the 30-day mortality and readmission of IHCA survivors based on their historical claims.

**Methods:**

National Health Insurance Research Database claims data, including 168,693 patients who had experienced IHCA at least once and 1,569,478 clinical records, were obtained to generate a data set for outcome prediction. We predicted the 30-day mortality/readmission after each current record (ALL-mortality/ALL-readmission) and 30-day mortality/readmission after IHCA (cardiac arrest [CA]-mortality/CA-readmission). We developed a hierarchical vectorizer (HVec) deep learning model to extract patients’ information and predict mortality and readmission. To embed the textual medical concepts of the clinical records into our deep learning model, we used Text2Node to compute the distributed representations of all medical concept codes as a 128-dimensional vector. Along with the patient’s demographic information, our novel HVec model generated embedding vectors to hierarchically describe the health status at the record-level and patient-level. Multitask learning involving two main tasks and auxiliary tasks was proposed. As CA-mortality and CA-readmission were rare, person upsampling of patients with CA and weighting of CA records were used to improve prediction performance.

**Results:**

With the multitask learning setting in the model learning process, we achieved an area under the receiver operating characteristic of 0.752 for CA-mortality, 0.711 for ALL-mortality, 0.852 for CA-readmission, and 0.889 for ALL-readmission. The area under the receiver operating characteristic was improved to 0.808 for CA-mortality and 0.862 for CA-readmission after solving the extremely imbalanced issue for CA-mortality/CA-readmission by upsampling and weighting.

**Conclusions:**

This study demonstrated the potential of predicting future outcomes for IHCA survivors by machine learning. The results showed that our proposed approach could effectively alleviate data imbalance problems and train a better model for outcome prediction.

## Introduction

### Background

In the United States, approximately 209,000 patients experience in-hospital cardiac arrest (IHCA) each year [1]. The rate of survival to hospital discharge is around 14%, and only 7% of IHCA patients could regain an independent life or a partially independent life [2]. In order to reduce the severe effect of IHCA on personal life or society, identifying measures to improve IHCA outcomes is crucial.

### Prior Work

Prognostic factors and prediction tools for survivors of IHCA and their neurologic outcomes have been identified in previous studies [3-8]. However, the evidence of an early warning system for predicting the mortality of IHCA survivors is limited. Current early warning scoring systems using physiologic track-and-trigger systems (TTSs) have been developed for identifying patients at risk for IHCA or other serious outcomes including mortality [9-11]. Most of TTSs rely on the routine observations of vital signs carried out by ward staff. Although many patients could be monitored with this approach, the quality of evidence underpinning the use of TTSs is poor. Specifically, most TTSs have low sensitivity, low positive predictive values, and high speciﬁcity [12,13]. In addition to the high mortality after IHCA, readmission after IHCA has a significant cost burden and is associated with comorbidities. Predicting readmission events provides the chance for appropriate interventions and reducing health care costs, including further readmission [14,15].

### Our Study

Here, we first extracted the IHCA cohort from the National Health Insurance Research Database (NHIRD). We assessed their risk based on historical electronic health records (EHRs) in the NHIRD. To provide a long enough window for clinical intervention, we used the 30-day mortality and readmission after IHCA as our prediction targets. In contrast to TTSs, EHRs are prepared by physicians, and they contain several important medical information, including the diagnosis and management of patients. To achieve a better performance, we developed a novel deep learning model, hierarchical vectorizer (HVec), to analyze the patients’ historical EHRs and predict mortality and readmission. This study aimed to demonstrate that with the proper data set and learning strategies, we can predict the outcome of IHCA patients based on their historical claims and help clinicians design more effective intervention programs.

## Methods

### Data Collection

This study was approved by the Institutional Review Board of National Taiwan University Medical College. The IHCA cohort extracted from the NHIRD consisted of 168,693 patients who had at least one IHCA event over 9 years (between January 1, 2002, and December 31, 2010). The Taiwan National Health Insurance program is the only health insurance scheme in Taiwan and covers up to 99.99% of Taiwan’s population [16]. The NHIRD contains all health records in inpatient and outpatient settings (clinic or emergency department); however, the records cannot be specifically linked to each patient. International Classification of Disease, 9th Revision (ICD-9) was used during the study period for diagnosis and medical procedures. The NHIRD contains medical information, including gender, age, diagnosis, medical procedure, operation, medication, laboratory test, care site, discharge status, and cost of each hospital visit. Laboratory test results and bedside information, including vital signs, blood pressure, and physical examination, are not included in the NHIRD.

**Figure 1:**
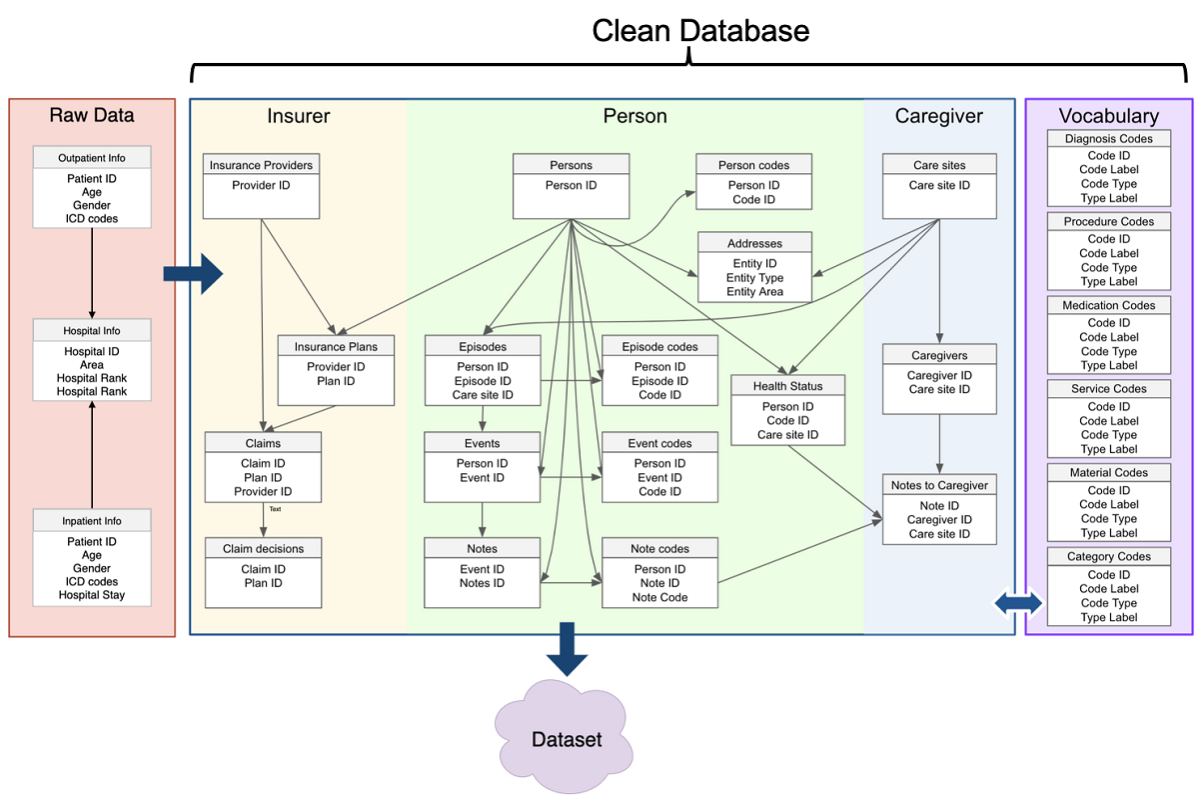
ETL process for converting raw NHIRD data into the data set. The raw data are extracted, transformed, and loaded into the cleaned database after cohort selection and eliminating the invalid data. ETL: extract, transform, load; ICD: International Classification of Disease; NHIRD: National Health Insurance Research Database.

The IHCA population was defined by inpatient records with the ICD-9 procedure codes 99.60 (cardiopulmonary resuscitation, not otherwise specified) and 99.63 (closed-chest cardiac massage) [17]. We used the extract, transform, and load (ETL, see Figure 1) procedure to process raw data into a clean database by eliminating records with missing or invalid information. Raw data in the cleaned database were re-grouped into three major categories (insurer, person, and caregiver) to improve data organization. In addition, vocabulary tables were constructed based on extracted concepts that were used in the raw data.

### Experimental Data Set

The 168,693 patients in the data set were split into three data sets: train, validation, and test. The training data set (70% of the data set) was used to train each model. The remaining 30% of patients were split between the validation and test data set evenly to tune the hyperparameters (Table 1) and evaluate model performance, respectively.

For comparison, we trained two single-task models for both mortality and readmission. Person upsampling and event weighting were only performed on the training data set so as not to affect the distribution of the validation and test data set. The F1 score and area under the receiver operating characteristic (AUROC) were our main evaluation metrics.

**Table 1:** Hyperparameter settings.

Hyperparameter	Value
Visit embed size	200
RNN^a^ output size	128
Learning rate	1e-3
Dropout rate	0.5
l2_weights	0.01
Code embed size	128

^a^RNN: recurrent neural network.

### Input Features

In the retrospective review of the data set, each person may have multiple clinical records (inpatient/outpatient visit) to the hospital within a 9-year period. Each clinical record was set as a unit of analysis. For each clinical record, information was extracted and grouped into input and target features (see Tables 2 and 3). The input features consisted of five major groups described as follows:

1. Medical records consisted of five types of codes, and all codes in the clinical records were mapped to over 400,000 clinical concepts. The health status of the patient in the current record can be determined from this group.
2. Demographics included the age, gender, and information of the targeted patients.
3. Care site information included information of the clinical institution where the patient received treatment.
4. Record statistics provided information on the length (by day) of the record, the number of codes in each International Classification of Disease (ICD) code category, and the total monetary cost involved. This could help the model estimate the severity of the patient’s disease at the record level.
5. Historical information described previous hospital stays and admissions. It was used to estimate the overall health status of the patient in our model.

**Table 2:** Summary of the input features of the model.

Group and feature name		Feature description	Dimension
**Medical records**			
	Diagnosis	Diagnosis codes from the health record	128
	Procedures	Procedure codes from the health record	128
	Meds	Medication codes from the health record	128
	Tests	Lab test codes from the health record	128
	Other	Other codes from the health record	128
**Demographics**			
	Claim type	Inpatient or outpatient	2
	Age	Age at the event	1
	Gender	Male or female	2
**Care site information**			
	Care site type	Type of site (public, corporate, or private)	21
	Care site specialization	Medical center, community hospital, district hospital, regional hospital, or clinic	5
	Care site rank	Rank of the care site	17
**Record statistics**			
	Hospital stay	Duration of current hospital stay	1
	Total cost	Monetary cost of each of the five ICD^a^ codes	5
	Total count	Counts of each of the five ICD codes	5
**Historical information**			
	Past hospitalization duration	The number of days a person spends in the hospital within 3, 6, 12, and 24 months	4
	Past admission count	The number of times a person is admitted to the hospital within 3, 6, 12, and 24 months	4

^a^ICD: International Classification of Disease.

**Table 3:** Summary of the prediction targets.

Group and feature name		Feature description	Dimension
**Main target**			
	Mortality	Whether this event would lead to another mortality event in (within 1 to 30) days	1
	Readmission	Whether this event would lead to another readmission event in (within 1 to 30) days	1

### Targets

A threshold of 30 days was set to predict whether a person would die or readmit within 30 days. Mortality was defined when the patient had an inpatient or outpatient record of mortality or was discharged under critical condition following IHCA. For readmission, whether the patient is readmitted within 1 to 30 days from hospital discharge was predicted. In contrast to other mortality studies, records with mortality (0-day mortality record) were excluded in our study. The main purpose of this strategy was to reduce the “leakage” of features in these records. Our initial results showed that the features of mortality records usually contain information (eg, respiratory failure) explicitly indicating patient mortality. Indeed, these features are significant factors for predicting mortality. However, such cases are not beneficial to our model as the severe condition of these patients makes it hard to treat them with any intervention. Moreover, the high degree of correlation of these features and mortality would cause the model to rely on them and underestimate other potential predictive factors. In order to avoid leakage and let the model focus on other predictive factors, mortality records were set as the negative class, and previous records of mortality (within 30 days) were the positive class.

**Figure 2:**
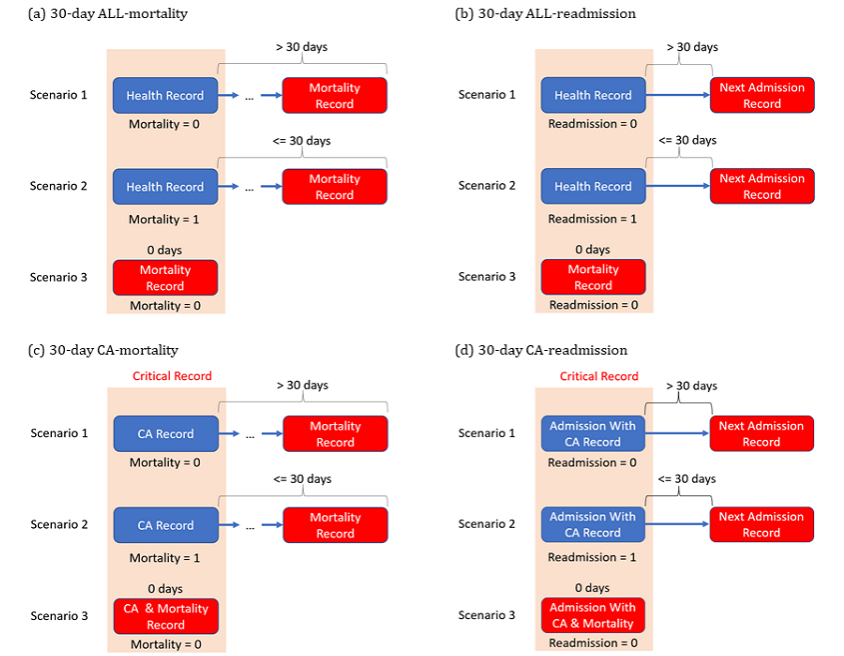
ALL/CA-mortality and ALL/CA-readmission. 3 scenarios of 30-days mortality & readmission after cardiac arrest. Events in red are the outcomes we want to predict. ALL-mortality: 30-day mortality after all records; ALL-readmission: 30-day readmission after all records; CA: cardiac arrest; CA-mortality: 30-day mortality after cardiac arrest records; CA-readmission: 30-day readmission after cardiac arrest records.

In clinical practice, the 30-day outcomes of patients after IHCA and discharge from hospitalization are of great interest. The 30-day mortality or readmission after cardiac arrest (CA; CA-mortality/CA-readmission) is a subset of 30-day mortality or readmission. In the rest of the paper, ALL-mortality/ ALL-readmission will be used to represent the 30-day mortality or readmission for all records. CA-mortality/CA-readmission refers to the 30-day mortality or readmission following CA (Figure 2).

ALL-mortality/ALL-readmission is more common than CA-mortality/CA-readmission as most people would not survive after the first CA event. This means that when we want to predict the future outcome of a recovered CA patient, we do not have enough positive cases for analysis.

### Hierarchical Vectorizer (HVec)

**Figure 3:**
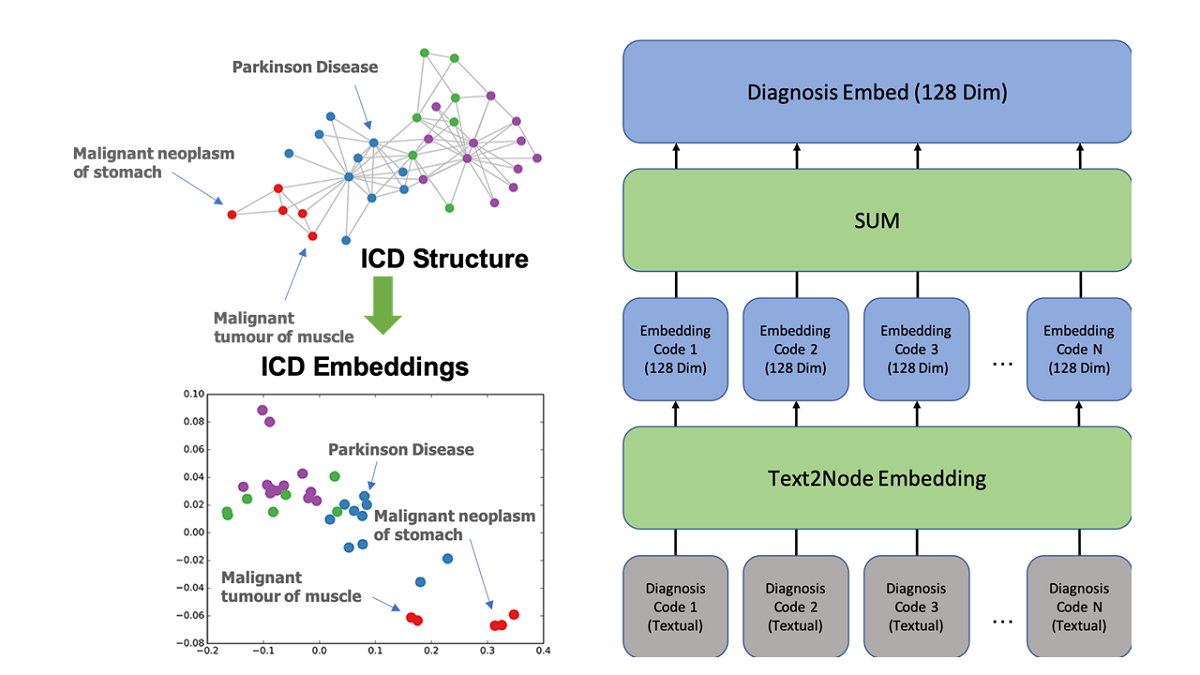
Example of Text2Node embedding [18] and code embedding for diagnosis codes in a clinical record. Dim: dimension; ICD: International Classification of Disease.

Each record was constructed into a 707-dimension vector for further training. Based on Table 1, all features except for ICD code features (textual features) can be vectorized with one-hot encoding. The features of ICD codes were extracted directly from the health record. A medical knowledge embedding system called Text2Node was used to embed the textual features into vectors [18]. Each of the five categories of ICD codes could contain many ICD codes from a single record, and all codes were added together as a single code for a given category (see Figure 3 as an example). Trained from a substantial medical knowledge database, Text2Node can effectively transform the textual medical concepts into a latent space while preserving the relationship of similar concepts.

**Figure 4:**
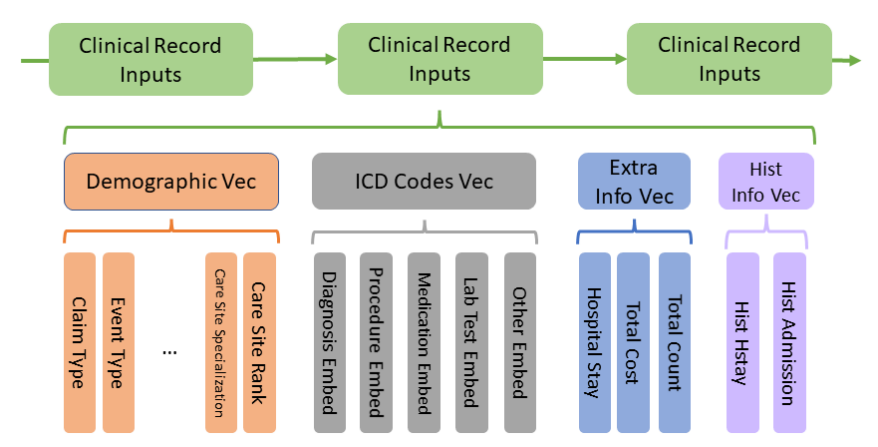
Feature concatenation to generate clinical record vectors for time series analysis. ICD: International Classification of Disease; Vec: vector.

For each clinical record, by concatenating all feature vectors into group vectors hierarchically (see Figure 4), the clinical record vector was obtained. After sorting each clinical record vector according to the date, time series techniques were used to train a model to predict the outcomes of each record.

### Model Architecture

**Figure 5:**
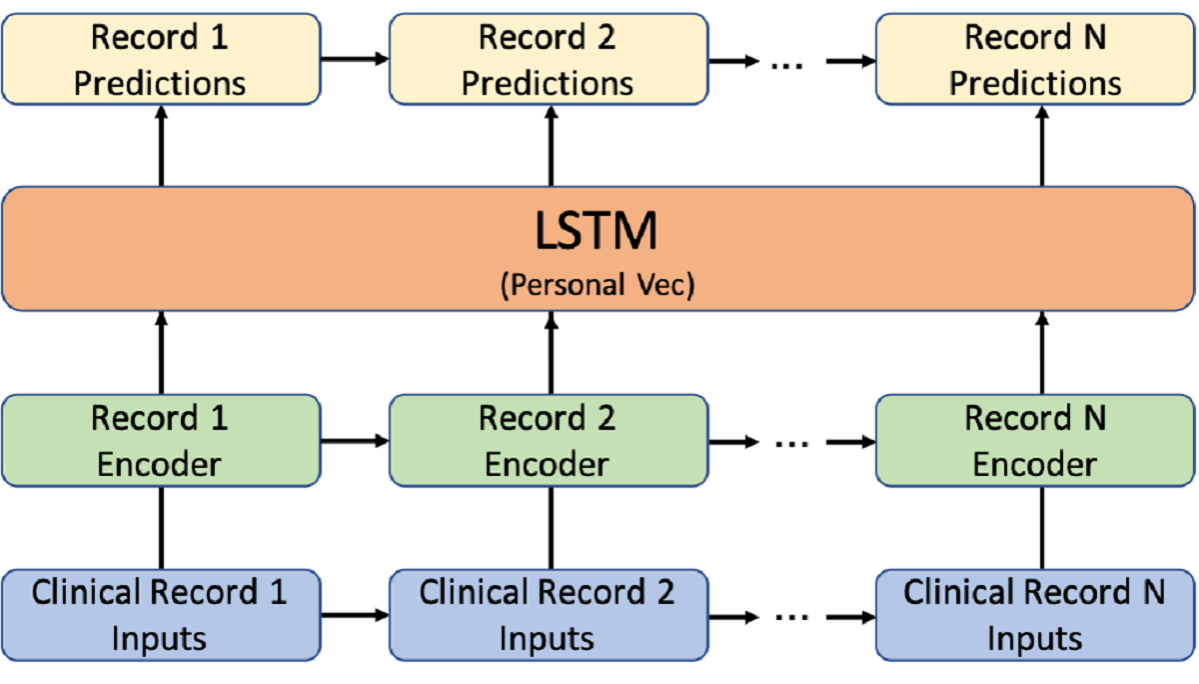
HVec model using LSTM networks. HVec: hierarchical vectorizer; LSTM: long short-term memory; Vec: vector.

Deep recurrent neural networks (RNNs) have been proven to be a powerful tool for predicting time series data. In clinical research, the use of RNNs, especially long short-term memory (LSTM), for clinical prediction has been widely investigated [19-21]. Inspired by Choi’s work [19], we proposed an HVec model using LSTM networks (Figure 5). In this framework, the record encoder was a fully-connected layer that generated the record embedding for each clinical record independently. The record embedding was a latent vector that contained all the information representing the current clinical record. This latent vector was used as the input of the LSTM to update the person vector (ie, patient status). This person vector was then used to predict our targets.

However, most previous studies were based on a relatively balanced data set. As we pointed out earlier, the distribution of CA-mortality/CA-readmission is extremely imbalanced. Training LSTM networks with an extremely imbalanced data set is always challenging because, without a carefully designed training strategy, the model could be biased (ie, predicting the negative class for all records). In order to address this problem, two different training strategies have been proposed to alleviate model bias during the training step:

1. The multitask learning framework was introduced to combine several related learning tasks to regularize the gradient and alleviate data imbalance problems during training.
2. At the person level, CA-mortality/CA-readmission records were upsampled, and at the record level, higher weights were assigned to CA records.

### Multitask Learning

**Figure 6:**
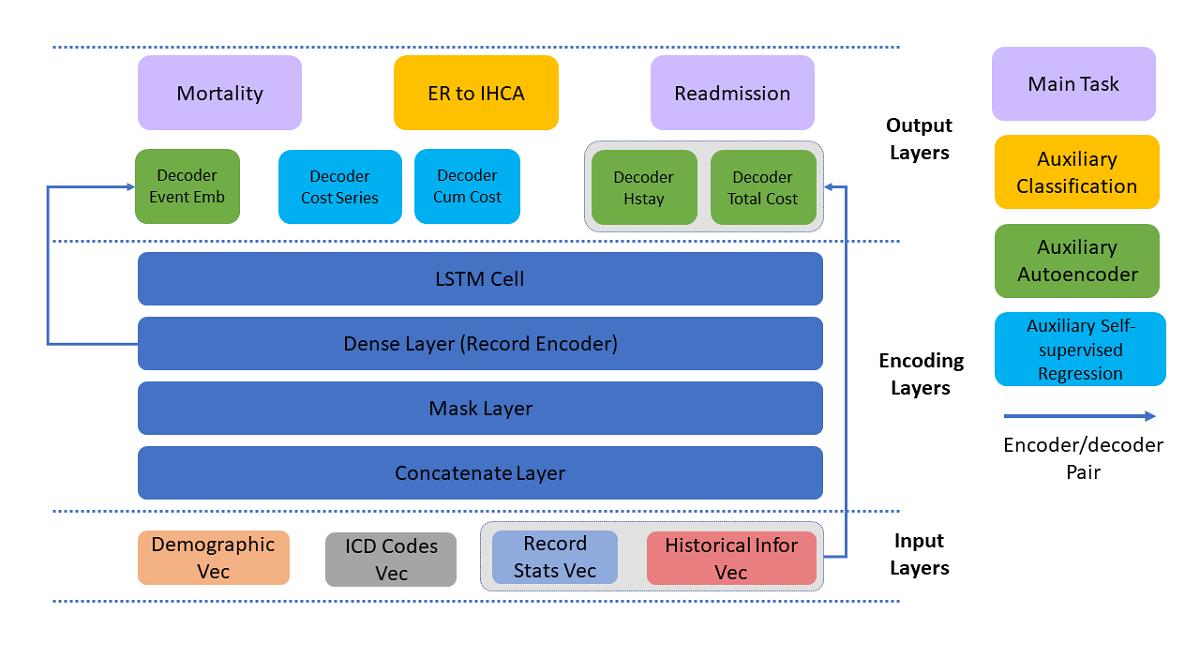
Multiple outputs for multitask learning. Cum: cumulative; Emb: embedding; ER: emergency room; Hstay: hospital staying; ICD: International Classification of Disease; IHCA: in-hospital cardiac arrest; Infor: information; LSTM: long short-term memory; Stats: statistics; Vec: vector.

The multitask learning framework was proposed, and several auxiliary related tasks were added to the HVec outputs (Figure 6). In this framework, the main functions were mortality and readmission. Here, instead of dividing each main task output into ALL-mortality/ALL-readmission and CA-mortality/ CA-readmission independently, an output was considered to cover both because the latter is a subset of the former. Although the distribution was different, they still can be achieved simultaneously with the proposed person upsampling and CA record weighting (described in the next section). Inspired by a previous study [22], three auxiliary autoencoder tasks were introduced to help the model learn the embeddings (Figure 6). Two self-supervised regression tasks were also introduced to allow the embedding to “memorize” the current cost and predict the future cost. Furthermore, another classification task was implemented to predict whether a record is an IHCA record considering that we observed the correlation of IHCA to mortality in the previous analysis.

To monitor the gradients of different tasks and regularize the learning process with auxiliary tasks, Theorem 1 was adopted from Du et al [23].

Theorem 1 given any gradient vector field G(θ^(t)^) = ∇_θ_𝓛 (θ) (1) to denote the main task and an arbitrary vector field V(θ^(t)^) to denote the gradient from another auxiliary task, the update strategy using:

θ^(t+1)^ := θ^(t)^ - α^(t)^ (G(θ^(t)^) + V(θ^(t)^) + max (0, cos(G(θ^(t)^), V(θ^(t)^))) **(2)**

with a proper can coverage to a local minimum.

Following this theorem, HVec can learn and converge with a large data set.

### Person Upsampling and CA Record Weighting

Although the gradients from different tasks can be monitored in multitask learning, with a heavily imbalanced data set (eg, CA-mortality), the auxiliary tasks may fail to regularize the main task. When we trained the HVec model, all records of a person were treated as a single sequential record and fed together into the model. Therefore, in each batch, the batch size was equal to the number of people in the batch. Compared with the number of all records, the number of CA records for each person was relatively rare. CA-mortality and CA-readmission were rare compared with ALL-mortality and ALL-readmission.

**Figure 7:**
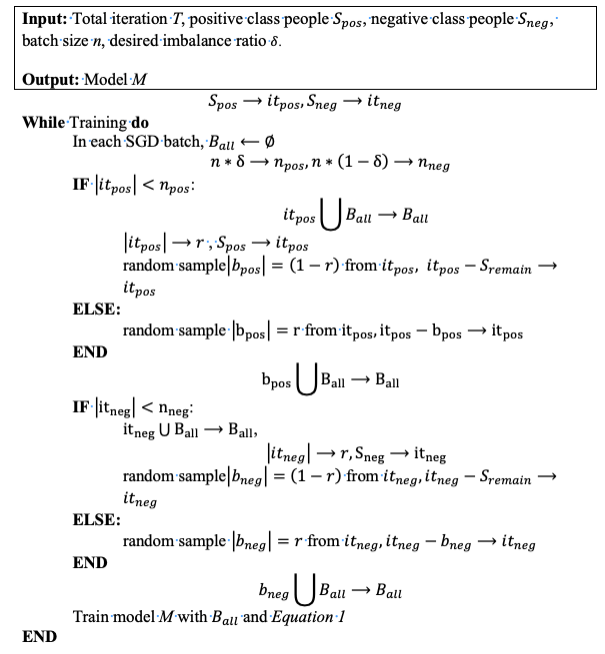
Person upsampling.

The weighting strategy [24] was proposed to solve this problem from two perspectives: at the person level, patients with CA-mortality/CA-readmission records were upsampled per batch (see Figure 7); at the record level, a higher weight was assigned to CA records to make the objective function more sensitive to CA-mortality/CA-readmission records. The upsampling of patients with CA-mortality/CA-readmission records can guarantee that at the person level, there are more CA-mortality/CA-readmission records [25].

Assigning a higher weight to CA records could also emphasize the CA records during training by modifying the loss functions accordingly. Considering *N*-loss functions {L_1_,…,L_N_} corresponding to auxiliary tasks, the loss function can be written as 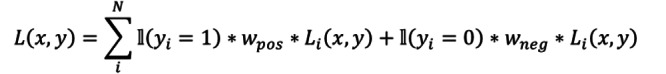 (3).

Where 
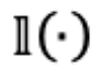
 (4) is a function that equals 1 if the statement in the bracket is true and otherwise 0; *w_pos_* and *w_neg_* are the positive and negative class weight, respectively. Combined with the gradient update strategy in equation 1, the HVec can learn from the extremely imbalanced data set effectively.

## Results

### Overview

**Figure 8:**
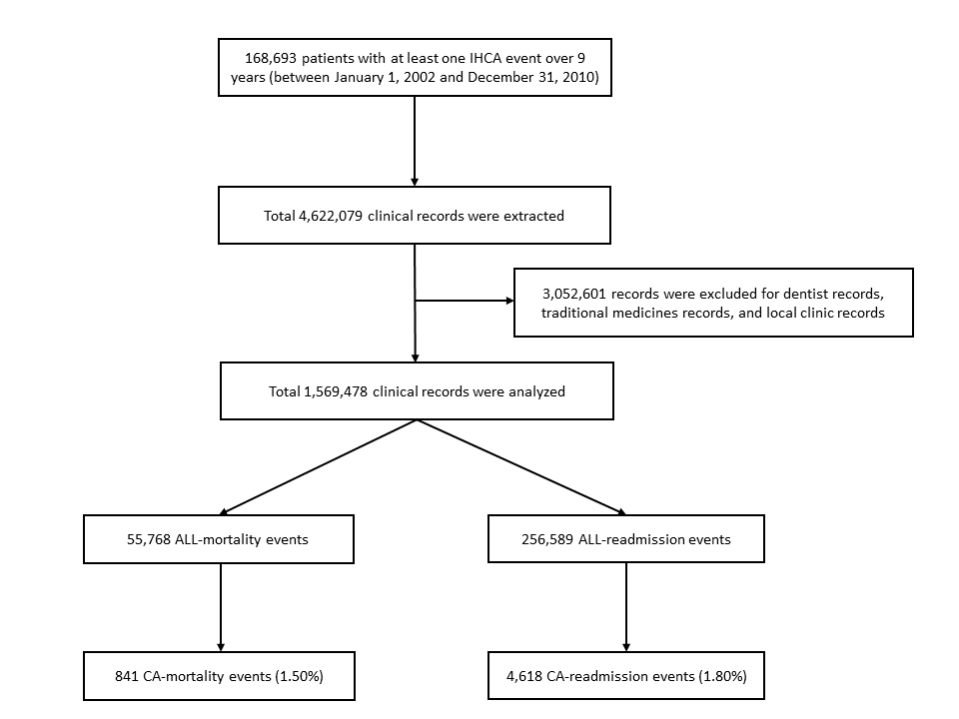
CONSORT diagram of the study cohort. ALL-mortality: 30-day mortality after all records; ALL-readmission: 30-day readmission after all records; CA: cardiac arrest; CA-mortality: 30-day mortality after CA records; CA-readmission: 30-day readmission after CA records; IHCA: in-hospital cardiac arrest.

A total of 168,693 patients and 4,622,079 clinical records were extracted from the NHIRD over 9 years, and 3,052,601 records (dentist records, traditional medicine records, or local clinic records) were excluded because these records were concentrated with repetitive conditions and mainly added noise to the machine learning models (Figure 8).

**Figure 9:**
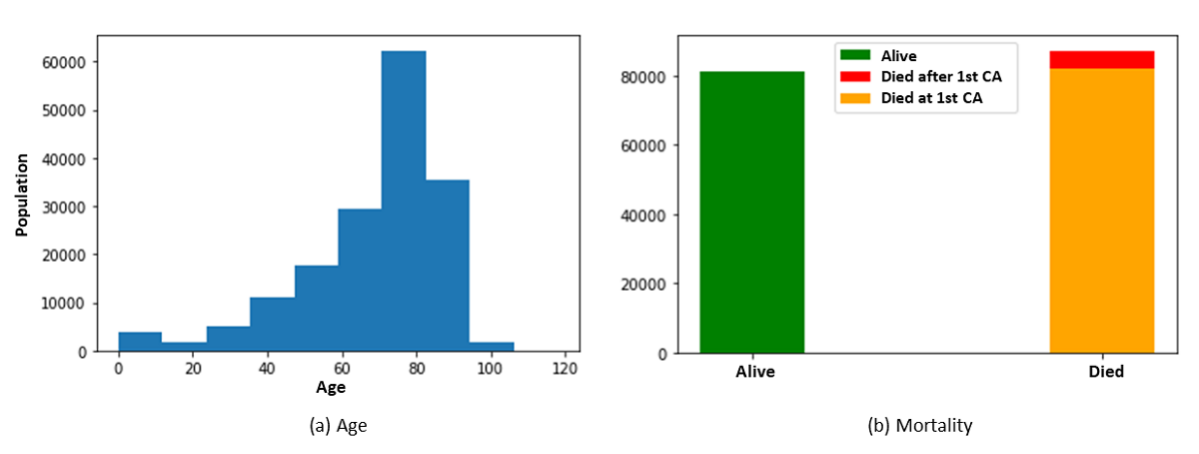
Age and mortality statistics of the data set. CA: cardiac arrest.

There were 1,569,478 clinical records in the cleaned database, including both inpatient and outpatient records, from 168,693 patients (mean number of records per person: 9.30, SD 10.90) who have experienced at least one IHCA event. The results indicate an imbalanced data set, where the most imbalanced task was CA-mortality with a ratio of 0.53%. The characteristics of the study population are summarized in Table 4. There were 173,345 IHCA records (11.04% of the total clinical records), and on average, there were 1.02 IHCA records for each person. The age of the patients in the data set ranged from 0 (newborn) to 118 years (mean age 68.66, SD 18.96 years), including 104,691 females and 64,002 males. Overall, 164,322 patients (97.4%) had CA only once, 4,174 patients (2.4%) had CA twice, and only 197 patients (0.2%) had CA more than twice. Death was recorded for 87,311 patients (51.75% mortality rate). Of these 87,311 patients, 82,225 patients died during their first hospitalization for CA (94.17%; Figure 9).

**Table 4:** Characteristics of the study population.^a^

Characteristics		Study population (N=168,693)
Age (years), mean (SD)		68.66 (18.96)
Gender (male), n (%)		64,002 (37.9)
Record number per person, mean (SD)		9.30 (10.90)
**Cardiac arrest frequency, n (%)**		
	1	164,322 (97.4)
	2	4174 (2.4)
	≥3	197 (0.2)
Mortality, n (%)		87,311 (51.75)

^a^Continuous variables are presented as the mean (SD), and categorical variables are presented as the number (percentage of the study population).

### Experiment 1: Single-Task Learning Versus Multitask Learning

In this experiment, person upsampling and event weighting were not applied. The model performance is summarized in Tables 5 and 6.

As shown in Table 5, multitask learning could improve the model performance for ALL-mortality and CA-mortality in terms of the AUROC and F1 scores. Based on single-task and multitask results, there was a relatively high improvement in performance for the extremely imbalanced CA-mortality task compared with the ALL-mortality task. However, the precision was relatively low due to the imbalance ratio, which also affected the F1 score. Moreover, the F1 score of CA-mortality was too low for real-life applications.

**Table 5:** Single-task and multitask learning performance for CA-mortality and ALL-mortality.^a^

Mortality	CA-mortality					ALL-mortality			
	AUROC^b^	F1	Precision	Recall	AUROC		F1	Precision	Recall
Single-task learning	0.658	0.014	0.010	0.024	0.663		0.130	0.101	0.180
Multitask learning	0.752	0.049	0.041	0.060	0.711		0.147	0.093	0.349

^a^CA-mortality: cardiac arrest mortality (30-day mortality after CA records); ALL-mortality: 30-day mortality after all records.

^b^AUROC: area under the receiver operating characteristic.

As shown in Table 6, the improvement in multitask learning for readmission prediction was not as significant as that for mortality prediction. Furthermore, in CA-readmission prediction, the F1 score and precision were decreased.

**Table 6:** Single-task and multitask learning performance for CA-readmission and ALL-readmission.^a^

Readmission	CA-readmission					ALL-readmission			
	AUROC^b^	F1	Precision	Recall	AUROC		F1	Precision	Recall
Single-task learning	0.847	0.214	0.162	0.315	0.872		0.554	0.424	0.801
Multitask learning	0.852	0.209	0.152	0.335	0.889		0.562	0.430	0.811

^a^CA-readmission: cardiac arrest readmission (30-day readmission after CA records); ALL-readmission: 30-day readmission after all records.

^b^AUROC: area under the receiver operating characteristic.

In this experiment, compared with single-task learning, multitask learning could achieve a better performance for ALL-mortality and ALL-readmission. However, multitask learning could not solve the extremely imbalanced data set.

### Experiment 2: Improving CA Prediction Performance

Experiment 1 showed that models had difficulties making good predictions based on the CA-mortality and CA-readmission data due to the extremely imbalanced data set. In this experiment, we demonstrated that by applying person upsampling and event weighting, we could further improve the performance for CA-mortality and CA-readmission without considerably affecting ALL-mortality and ALL-readmission. In our experiment, the upsampling rate indicates how many times upsampling was performed for the positive class (patients with CA-mortality or CA-readmission) in a batch, and an upsampling rate of 1 means we did not perform upsampling. The CA event weight indicates the loss weight *w_pos_* in equation 3, and we always set *w_neg_*=1. We used different upsampling rates and event weights in our experiments. Our results are summarized in Tables 7 and 8, including the previous results on single-task model performance (first row of each table).

Overall, compared with the 30-day mortality task, the imbalanced 30-day mortality task showed a larger increase in performance when applying balancing techniques. As shown in Table 7, after upsampling and event weighting, the models demonstrated improved performance for both ALL-mortality and CA-mortality. For CA-mortality, the F1 score was increased by 36.7% (from 0.049 to 0.067).

**Table 7:** Mortality models with their respective hyperparameter configuration and their performance in predicting both CA-mortality and ALL-mortality.^a^

Mortality				CA-mortality			ALL-mortality	
Upsampling rate	Event weight	Multitask	AUROC^b^		F1	AUROC		F1
1	1	No	0.658		0.014	0.663		0.130
1	1	Yes	0.752		0.049	0.711		0.147
10	1	Yes	0.808		0.064	0.728		0.155
10	5	Yes	0.802		0.067	0.726		0.158

^a^CA-mortality: cardiac arrest mortality (30-day mortality after CA records); ALL-mortality: 30-day mortality after all records.

^b^AUROC: area under the receiver operating characteristic.

Similar to ALL-mortality and CA-mortality, the AUROC and F1 score were increased for both ALL-readmission and CA-readmission by applying the two techniques. Based on the results in Table 8, a minor but consistent increase was achieved in CA-readmission prediction. However, there was no significant improvement in ALL-readmission prediction. This is because upsampling and event weighting would not greatly affect the learning process for a more balanced data set.

**Table 8:** Readmission models with their respective hyperparameter configuration and their performance in predicting CA-readmission and ALL-readmission.^a^

Readmission				CA-readmission			ALL-readmission	
Upsampling rate	Event weight	Multitask	AUROC^b^		F1	AUROC		F1
1	1	No	0.847		0.214	0.872		0.554
1	1	Yes	0.852		0.209	0.889		0.562
5	1	Yes	0.861		0.230	0.884		0.555
5	5	Yes	0.862		0.237	0.884		0.555

^a^CA-readmission: cardiac arrest readmission (30-day readmission after CA records); ALL-readmission: 30-day readmission after all records.

^b^AUROC: area under the receiver operating characteristic.

In summary, the results indicated that multitask learning, upsampling, and event weighting could effectively improve the model prediction performance for an imbalanced data set. We also showed that these techniques could be collectively used to achieve better results for an extremely imbalanced data set.

## Discussion

In this study, we constructed a large patient database that includes 9 years of EHRs for over 168,000 IHCA patients, which can be used for future IHCA-related research. In addition, we developed an HVec model (LSTM model) that uses a multitask learning strategy to predict the 30-day mortality and readmission.

The results showed that our model could successfully predict future mortality and readmission using EHR data for IHCA patients. We proposed the person upsampling and record weighting strategies to handle the extremely imbalanced data problem in this study. After applying these techniques, some improvements were achieved in CA-mortality and CA-admission prediction.

In contrast to other studies using deep learning models to predict another IHCA event after CA [26], our study focused on predicting the future outcomes of IHCA patients after discharge. To the best of our knowledge, this is the first study to predict mortality and readmission after IHCA events by machine learning. The model may serve as a surveillance system for those who experienced IHCA. Patients with a high risk of mortality or readmission in the near future could be identified and re-evaluated before discharge. This study also demonstrated the potential of another model for predicting future mortality and readmission after each record using previous EHRs (ALL-mortality/ALL-readmission). The model might help identify those with a high risk in inpatient and outpatient situations. However, mortality and readmission rates are different in the general population compared with the patients selected in this study. Using hospital EHRs with patients’ information in the NHIRD, we can construct a real-time alert system based on machine learning methods to predict the adverse events of IHCA survivors and improve their outcomes. Further prospective studies are needed to verify the utility of this system in the general population.

Several studies have reported models for predicting the outcomes of CA patients [8]. In a systematic review of current prediction models, the median AUROC value was 0.84 with an IQR of 0.80 to 0.89 [8]. For IHCA survivors, Chan et al. reported that the cardiac arrest survival post-resuscitation in-hospital (CASPRI) score could be used to predict favorable neurologic outcomes after discharge [3]. The AUROC of the CASPRI score was 0.80. Nanayakkara et al used deep learning models to predict the IHCA events of CA survivors, and the AUROC was 0.87 [26]. In our study, we encountered the difficulty of imbalanced data. Using proper learning strategies, we achieved comparable AUROC values (0.808 for CA-mortality and 0.862 for CA-readmission). However, the recall and precision rates were low in our study due to imbalanced data. This is a challenge we aim to resolve. When using historical medical records to predict outcomes, in many cases, the records contain information that may indicate the outcomes. For example, critical diagnosis and rescue medication are often associated with mortality. Including these types of information can facilitate training and give a high AUROC and F1; however, the model itself is of limited use. Mortality records were set as the negative class in our study to avoid overfitting. In further studies, the model may be improved by adjusting the threshold to optimize the trade-off between specificity and sensitivity.

In our HVec framework, we encoded each person’s EHRs in two levels of latent vectors (record-level and person-level) and ensured that the model learns both simultaneously using the unsupervised autoencoder strategy. The predictive results were promising with these latent vectors. The latent vectors in these two levels may be further explored to facilitate clinicians’ decision-making and provide better clinical interventions. In addition, the person vector may be used as a biomarker to evaluate the overall health status of a person beyond the health care setting. Along with some recently developed models such as Deep Patient [22] and MixEHR [27], we showed that the use of deep neural networks to extract information from EHRs might solve complex clinical research problems.

An imbalanced data distribution is common in clinical research, especially for disease-related predictions. In comparison with common diseases, many important diseases lack positive cases, making it difficult to train a good model. In a previous study [19], the authors attempted to address this problem by using a balanced, distributed data set and train a deep learning model with the balanced data set. Similarly, in this study involving CA-mortality and CA-readmission, we demonstrated that by carefully designing model learning strategies (eg, multitask learning and upsampling), some common problems in clinical research could be solved effectively with machine learning models.

This study has some limitations. First, the IHCA cohort was retrospectively extracted from the NHIRD. Further studies are needed to evaluate the efficacy of this model as an early warning system and determine how this system affects patients’ outcomes. Second, as our model was developed based on the NHIRD, the generalization of this model to other health insurance data sets is not proven. Third, each patient’s vital signs and laboratory data were not included in the analysis due to the study design. A combination of EHRs and patients’ clinical data may further improve model performance. Fourth, traditional machine learning methods have the limitation of interpretability. Specific risk factors for 30-day mortality/readmission were unknown in this study. In future studies, we plan to develop an explainable model and investigate specific predictive factors in the model.

In summary, our model showed good performance in predicting 30-day mortality and readmission after IHCA, which can help clinicians monitor CA patients' status better. We aim to provide more insights to clinicians with proactive intervention recommendations. Nevertheless, a challenge remains in the interpretative ability of the deep learning model. Our future work will mainly focus on the interpretative power of the model trained using EHRs.

## Abbreviations

AUROC: area under the receiver operating characteristic
CA: cardiac arrest
CASPRI: cardiac arrest survival post-resuscitation in-hospital
CPR: cardiopulmonary resuscitation
EHR: electronic health record
ETL: extract, transform, and load
HVec: hierarchical vectorizer
ICD: International Classification of Disease
IHCA: in-hospital cardiac arrest
LSTM: long short-term memory
NHIRD: National Health Insurance Research Database
OHCA: out-of-hospital cardiac arrest
RNN: recurrent neural network
TTS: track-and-trigger system
